# Molecular Characterization of HIV-1 CRF01_AE in Mekong Delta, Vietnam, and Impact of T-Cell Epitope Mutations on HLA Recognition (ANRS 12159)

**DOI:** 10.1371/journal.pone.0026244

**Published:** 2011-10-19

**Authors:** Estibaliz Lazaro, Luong Thu Tram, Pantxika Bellecave, Gwenda-Line Guidicelli, Guerric Anies, Huynh Hoang Khanh Thu, Marie Pillot Debelleix, Muriel Vray, Patricia Recordon-Pinson, Jean-Luc Taupin, Truong Thi Xuan Lien, Herve Fleury

**Affiliations:** 1 Laboratoire de Virologie, CHU de Bordeaux et CNRS-UMR 5234, Université Victor Segalen-Bordeaux 2, Bordeaux, France; 2 Pasteur Institute, Ho Chi Minh City, Vietnam; 3 Laboratoire d'immunologie et immunogénétique, CHU de Bordeaux, Bordeaux, France; 4 CNRS-UMR 5164, Université Victor Segalen-Bordeaux 2, Bordeaux, France; 5 Institut Pasteur, Paris, France; Jiangsu University, China

## Abstract

**Background:**

To date, 11 HIV-1 subtypes and 48 circulating recombinant forms have been described worldwide. The underlying reason why their distribution is so heterogeneous is not clear. Host genetic factors could partly explain this distribution. The aim of this study was to describe HIV-1 strains circulating in an unexplored area of Mekong Delta, Vietnam, and to assess the impact of optimal epitope mutations on HLA binding.

**Methods:**

We recruited 125 chronically antiretroviral-naive HIV-1-infected subjects from five cities in the Mekong Delta. We performed high-resolution DNA typing of HLA class I alleles, sequencing of Gag and RT-Prot genes and phylogenetic analysis of the strains. Epitope mutations were analyzed in patients bearing the HLA allele restricting the studied epitope. Optimal wild-type epitopes from the Los Alamos database were used as reference. T-cell epitope recognition was predicted using the immune epitope database tool according to three different scores involved in antigen processing (TAP and proteasome scores) and HLA binding (MHC score).

**Results:**

All sequences clustered with CRF01_AE. HLA class I genotyping showed the predominance of Asian alleles as A*11:01 and B*46:01 with a Vietnamese specificity held by two different haplotypes. The percentage of homology between Mekong and B consensus HIV-1 sequences was above 85%. Divergent epitopes had TAP and proteasome scores comparable with wild-type epitopes. MHC scores were significantly lower in divergent epitopes with a mean of 2.4 (±0.9) versus 2 (±0.7) in non-divergent ones (p<0.0001).

**Conclusions:**

Our study confirms the wide predominance of CRF01_AE in the Mekong Delta where patients harbor a specific HLA pattern. Moreover, it demonstrates the lower MHC binding affinity among divergent epitopes. This weak immune pressure combined with a narrow genetic diversity favors immune escape and could explain why CRF01_AE is still predominant in Vietnam, particularly in the Mekong area.

## Introduction

Since its introduction in humans, HIV-1 has undergone dramatic diversification, extensively spreading in all the continents and rapidly diversifying in Caucasian, African and Asian populations. The radial evolution of group M has led to multiple clades or subtypes (A1, A2, B, C, D, F1, F2, G, H, J and K), all of which are circulating in African populations. To date, 11 HIV-1 subtypes and 48 circulating recombinant forms have been described worldwide. HIV-1 subtype B predominates in the Caucasian populations of Europe and the Americas, HIV-1 subtype C in South Asia and CRF01_AE in South East Asia. In particular, a large number of HIV-1 recombinants between subtypes B and C (including CRF07_BC and CRF08_BC) are largely responsible for the current AIDS epidemic in China [1], [2]. The underlying reason why the distribution of the different HIV-1 subtypes is so heterogeneous and unbalanced is not clear. Viral lineage, defined as the founder effect, is certainly an important driver of HIV-1 evolution [3] but cannot explain all virus-clade-specific differences, which are also the consequence, at least in part, of the worldwide HLA distribution. The rapid spread of some subtypes and/or recombinants in Oriental populations could be due to the relative genetic homogeneity of these populations, which contrasts with the extreme genetic diversity in Africans [4], [5], [6].

HLA class I molecules play a critical role in defining the epitopes of cytotoxic T lymphocytes (CTLs), which are essential in host viral defense against HIV [7]. Large cohort studies have shown that certain HLA class I alleles correlate with HIV control, HLA B*57:01 being the most consistently recognized association with immune control of infection [8], [9], [10], [11]. Moreover, homozygosity is associated with faster progression to AIDS, suggesting that heterozygosity at each HLA class I allele allows the presentation of a greater range of epitopes and a more effective control of HIV replication [12], [13].

Mutations affecting HLA-restricted epitopes may lead to immune escape if they impact on epitope antigen processing, HLA binding or TCR recognition. Several studies have identified a growing number of epitope mutations leading to immune escape, especially in Caucasian populations infected with subtype B or African populations infected with subtype C [14], [15], [16].

In Vietnam, we and others have demonstrated that CRF01_AE is widely predominant, but little is known about the impact of epitope mutations observed among Asian HIV-1 infected patients on CTL recognition [17], [18], [19], [20].

The aim of this study was (i) to describe HIV-1 subtypes circulating among antiretroviral (ART)-naive patients in an unexplored area, the Mekong Delta, Vietnam; (ii) to analyze the polymorphism of Gag and RT-Prot sequences; and (iii) to study the impact of amino acid divergences within CRF01_AE epitopes on antigen processing and HLA binding among this host Vietnamese population.

## Methods

### Ethical statement

This study was approved by the Ethics Committee of the Vietnamese Ministry of Public Health and was conducted in accordance with the set guidelines for research. All patients provided their written informed consent for the collection of the samples and their subsequent analysis.

### Population and samples

**Figure 1:**
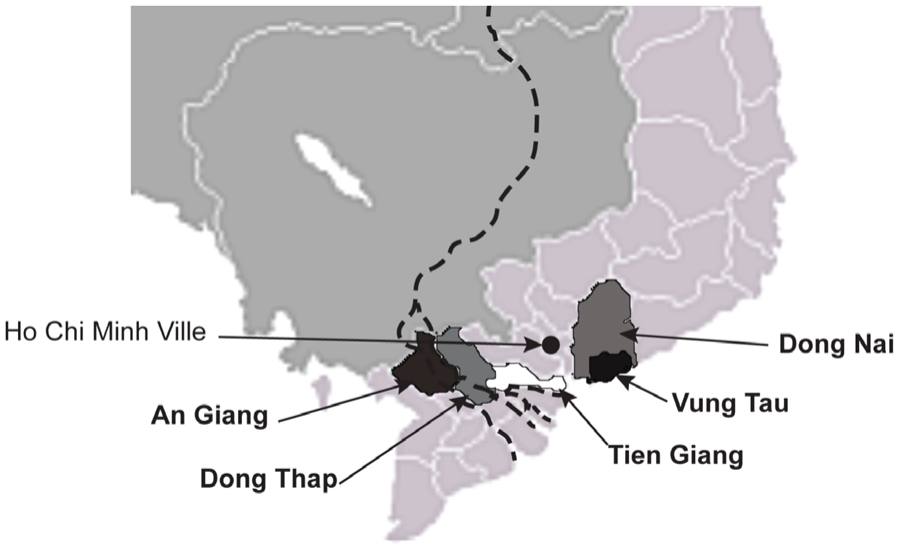
Vietnamese cities of Mekong Delta taking part to the study.

From June to October 2008, 125 chronically HIV-1-infected individuals including 80 men and 45 women were recruited from 5 centers for preventive medicine located in the provincial cities of the Mekong Delta (Vietnam): Dong Nai, Vung Tau, Tien Giang, An Giang and Dong Thap (**Figure 1**). Most (95.2%) of these individuals are drug users or sexually transmitted disease patients. The median age was 30 (20–50) years. All patients except for 3 individuals who were receiving treatment are antiretroviral therapy (ART)-naïve.

Blood samples were collected from each patient using ethylenediaminetetraacetic acid (EDTA) tubes. CD4 T lymphocytes were counted with a FACScan flow cytometer (BD Biosciences, San Jose, CA). The plasma samples were separated and stored at −80°C until use.

The median CD4+ cell count in our study population was 147 cells/µl (1–1032) and the plasma viral load was unknown. Clinical staging was as follows according to the WHO classification: 33% stage I, 27% stage II, 35% stage III, 5% stage IV.

### HLA class I typing

Genomic DNA was extracted from the frozen white blood cell pellets using the MagNA Pure automatic system (Roche) according to the manufacturer's specifications, and quantitated by UV optical density measurement. Intermediate-to-high resolution was performed by reverse Polymerase Chain Reaction-Sequence Specific Oligonucleotide (PCR-SSO) hybridization using the Luminex® flow beads LabType® assay (InGen, Chilly-Mazarin, France) for the A, B and Cw loci. Allelic ambiguities were solved with PCR-Sequence Specific Primer (SSP) amplification using Olerup assays (BioNoBis, Montfort L'Amaury, France). The manufacturers' recommendations were strictly followed. Allele assignment was performed by comparison with the official nomenclature of April 2010 and validated by the WHO committee for HLA system factors [21].

### Allele frequencies and linkage disequilibrium

Allele and haplotype frequencies as well as linkage disequilibrium between all pairs of HLA loci were obtained with the HLA Frequency Analysis Tool of the Los Alamos HIV Immunology Database (http://www.hiv.lanl.gov/content/immunology/tools-links.html). Frequency data for other populations were obtained from previous studies: Kinh Vietnamese [22], Chinese and European [23].

### PCR amplification of HIV-1 Prot, Gag and RT, and sequencing

Viral RNA was extracted from 200 µl of plasma samples using the MagNA Pure LC Total Nucleic Acid Isolation-High Performance kit with the MagNA Pure LC system ((Roche Diagnostics, Mannheim, Germany). HIV regions were amplified by RT-PCR using the Titan One Tube kit (Roche Diagnosis, Mannheim, Germany) followed by nested PCR using the Amplitaq Gold with GeneAmp Kit (Applied Biosystem, Foster City, CA). The protease gene was amplified and sequenced according to the ANRS procedure and Gag gene as previously described [18]. The whole RT gene was obtained by using 3 sets of primers, corresponding to polymerase (outer primers: MJ3 5′-AGTAGGACCTACACCTGTCA-3′ and MJ4 5′-CTGTTAGTGCTTTGGTTCCTCT-3′; inner primers: A(35) 5′-TTGGTTGCACTTTAAATTTTCCCATTAGTCCTATT-3′ and NE1(35) 5′-CCTACTAACTTCTGTATGTCATTGACAGTCCAGCT-3′), thumb-connection (outer primers: 02TCRT5 5′-GGATGGAAAGGATCACCAGCAAT-3′ and 02TCRT3 5′-TGATTTGTTGTCTCAGTTAGGGAA-3 inner primers: 02TCnes5 5′-TGGATGGGATATGAACTCCATCCTGA-3′ and 02TCnes3 5′-TTAGCTGCCCCATCTACATCG-3′ and RNase H domains (outer primers: 02RNRT5 5′-AAATATGCAAAAAGGAGGTCTGC-3′ and 02RNRT3 5′-CAGTCTACTTGTCCATGCATGGC-3′; inner primers: 02RNnes5 5′-GAAACATGGGAAGCATGGTGGATGGAGTA-3′ and 02RNnes3 5′-TCTTCTTGGGCTTTATCTATGCCATCTA-3′. PCR products were sequenced on both strands using a CEQ DTCS Quick Start kit on an automated sequencer Beckman CEQ 2000 DNA analyzer system (Beckman Coulter, Fullerton, CA) as previously described [18]. Genotypic resistance was interpreted with the ANRS algorithm v18.

### Phylogenetic analysis

The derived nucleotide sequences of RT, Prot and Gag regions were aligned by the Clustal W 1.74 alignment program with known reference strains of M, N and O pooled from the HIV Database (http://www.hiv.lanl.gov/). Phylogenetic trees were inferred by the neighbor-joining method from matrix distances calculated after gap stripping of alignments, according to a Kimura two-parameter algorithm. The circular trees were obtained using the on-line tool ITOL (http://itol.embl.de/) [24].

GenBank accession numbers for the sequences reported in this study are HQ542709 to HQ542803, HQ542613 to HQ542708 and HQ542545 to HQ542612 for RT, Prot and Gag sequences respectively.

### Sequence alignment and consensus

Protein sequence analyses were performed using tools available at the Institut de Biologie et Chimie des Protéines (IBCP) Network Protein Sequence Analysis (NPSA) website (http://npsa-pbil.ibcp.fr) [25]. Multiple-sequence alignments of protein sequences obtained from patients were performed with ClustalW using default parameters. A primary consensus Mekong sequence was generated for Gag, RT and Prot and was used for alignment with consensus B and HxB2 strains. Identity was determined as a percentage of strictly conserved amino acids between the two sequences compared.

### Immune recognition tools

The immune epitope database (www.immuneepitope.org) was used to predict T-cell epitope recognition. The different steps involved in the MHC class I antigen presentation pathway were evaluated by three scores: the proteasomal score reflects the efficiency of antigen-processing by the total amount of cleavage site usage releasing the peptide C-terminus; the TAP score predicts transporter associated with antigen processing (TAP) transport by estimating the binding of a peptide or its N-terminal prolonged precursors to TAP, with highest affinity for a peptide meaning the highest transport rates; the MHC score defines the epitope affinity for the MHC molecule. All scores are logarithmic values with higher values indicating higher predicted efficiency.

### Statistical analysis

Comparisons between the two groups were performed with the paired t-test using GraphPad Prism version 4.00 for Macintosh (GraphPad Software, San Diego, CA, USA). A p≤0.05 was considered as statistically significant.

## Results

### Sequence analysis

**Figure 2:**
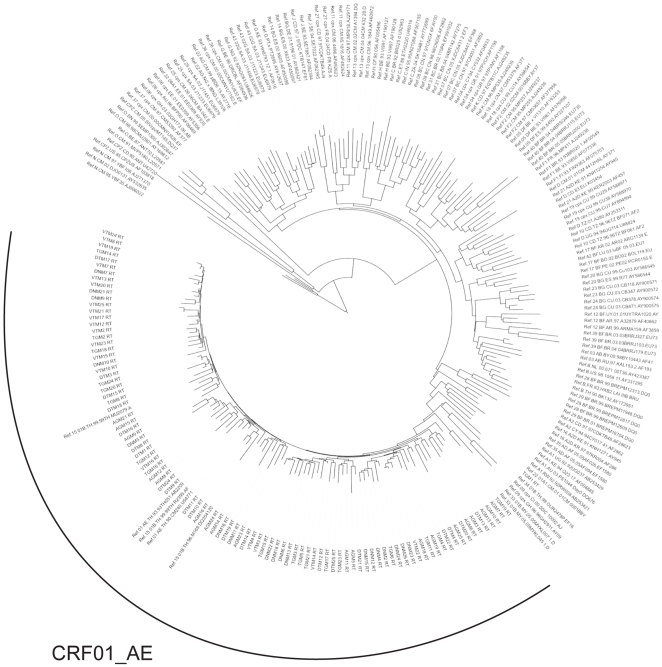
Phylogenic analysis of RT sequences of 125 Vietnamese HIV-1 patients.

Sequence genotyping was performed for 105 samples. All RT, Prot and Gag sequences clustered with subtype CRF01_AE (depicted for RT gene in **Figure 2**, data not shown for the other genes). The detailed analysis of these sequences allowed us to define (i) general polymorphisms of CRF01_AE sequences, (ii) specific polymorphisms located in intra-epitopic regions, (iii) drug resistance mutations.

#### General polymorphisms

A CRF01_AE Vietnamese natural consensus including polymorphisms present in more than 50% of the sequences was established for Gag, RT and Prot proteins. The percentage of homology found between the Mekong consensus reference and the B or CRF01_AE consensus reference (as defined in Los Alamos database) is shown in **Table 1**. Our Mekong consensus sequence was in agreement with the CRF01_AE consensus observed in untreated Southeast Asian patients and described in previous studies, with a percentage of homology above 98%. The percentage of homology between the Mekong and B consensus sequences was 93.2%, 92.9% and 85.1% for RT, Prot and Gag amino acid sequences, respectively (92.5%, 90.9% and 85.3% respectively with the HxB2 strain).

**Table 1:** Percentage of homology between CRF01_AE Vietnamese consensus sequences of RT, Prot and Gag aligned with the consensus CRF01_AE or B strain.

	Mekong RT	Mekong Prot	Mekong Gag
**CRF01_AE consensus**	99.1%	99.0%	84.6%
**B consensus**	93.2%	92.9%	85.1%

#### Amino Acid substitutions located in intra-epitopic regions

**Figure 3:**
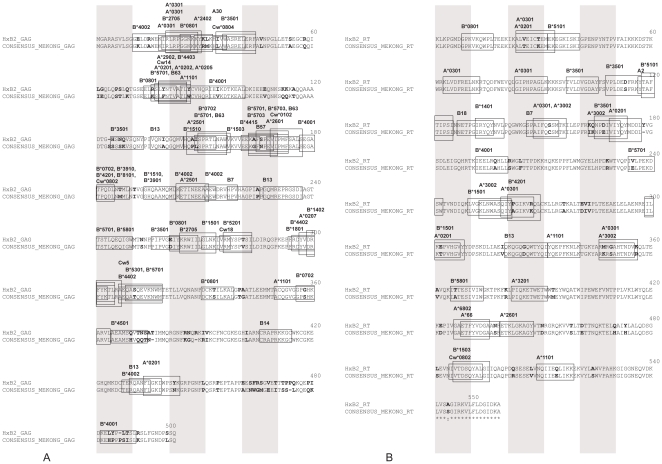
Comparison of Gag (A) and RT (B) Vietnamese consensus sequences with HxB2 strains. Optimal CTL epitopes are highlighted by boxes. HLA restriction is indicated on the corresponding epitopes. Amino acid substitutions are indicated in bold. Shaded vertical bars separate blocks of 10 amino acids.

Using this consensus sequence, we focused our analysis on polymorphisms present in optimal CTL epitopes as defined in the Los Alamos database. **Figure 3** depicts the location and HLA restriction elements of CTL epitopes on Gag and RT sequences on HxB2 aligned with the CRF01_AE Vietnamese consensus sequence. As there are only very few differences between Mekong and whole CRF01_AE consensus sequences and these differences are not relevant in terms of HLA binding predictions, the consensus amino acid of CRF01_AE was not added in the figure.

A detailed analysis of amino acid substitutions in epitopic regions was assessed for RT, Gag and Prot sequences and compared to the reference strain HxB2. A high number of amino acid polymorphisms were detected in optimal CTL epitopes as defined in the Los Alamos database. The analysis was accurately performed for 106 optimal CTL epitopes displayed by Gag, RT and Prot proteins. The proportion of divergent epitopes was 45.2% (19/42) in RT-Prot protein and 57.8% (37/64) in Gag protein. Thirteen RT epitopes presented more than 60% of identity with HxB2 epitopes, while 20 RT epitopes exhibited less identity including 13 highly divergent epitopes, *i.e.* epitopes with conserved divergence among all patient sequences (proportion of 60.6% divergent RT epitopes). With regard to Gag, more than 60% of identity between Mekong and HxB2 epitope sequences was observed for 20 epitopes. Among the 44 epitopes with low identity, 24 were highly divergent (corresponding to a proportion of 68.7% of the Gag epitopes).

#### Drug resistance mutations

According to the international list of surveillance drug resistance mutations (SDRMs) updated in 2009 [26], we identified some drug resistance mutations (DRMs) from 7 HIV-1 infected individuals. None of them had received any antiretroviral treatment. The DRMs in the RT are listed in **Table 2**. These DRMs are demonstrated to be unrelated to polymorphism, and are associated with nucleoside reverse transcriptase inhibitors (NRTIs) (184V, 65R) and non-nucleoside reverse transcriptase inhibitors (NNRTIs) (98G, 103N, 106I, 181C, 181I, 188V, 190A, 230L).

**Table 2:** Drug resistance mutation (DRM) in RT sequences according to the international list of surveillance drug-resistance mutations (SDRMs) updated in 2009 [26].

Sample ID	RT DRMs	NRTI and NNRTI resistance
DN-M3	230L	NVP, EFV
DT-M8	181C	NVP, EFV
DT-M9	65R, 181C	TDF, NVP, EFV
DT-M12	103N, 190A	NVP, EFV
DT-M15	184V, 181I	3TC, NVP, EFV
DT-M24	103N	NVP, EFV
DT-M19	98G, 106I, 181C, 188V	NVP, EFV, ETV

NVP = nevirapine, EFV = efavirenz, TDF = tenofovir, 3TC = lamivudine, ETV = etravirine.

In addition, the protease-coding region carried minor resistance mutations to protease inhibitors (PIs) (for example 36I) reflecting natural polymorphism in non-B subtypes. The major DRM 46I was observed in one patient but this mutation can be considered as a polymorphism in CRF01_AE [26].

### HLA Class I typing

**Figure 4:**
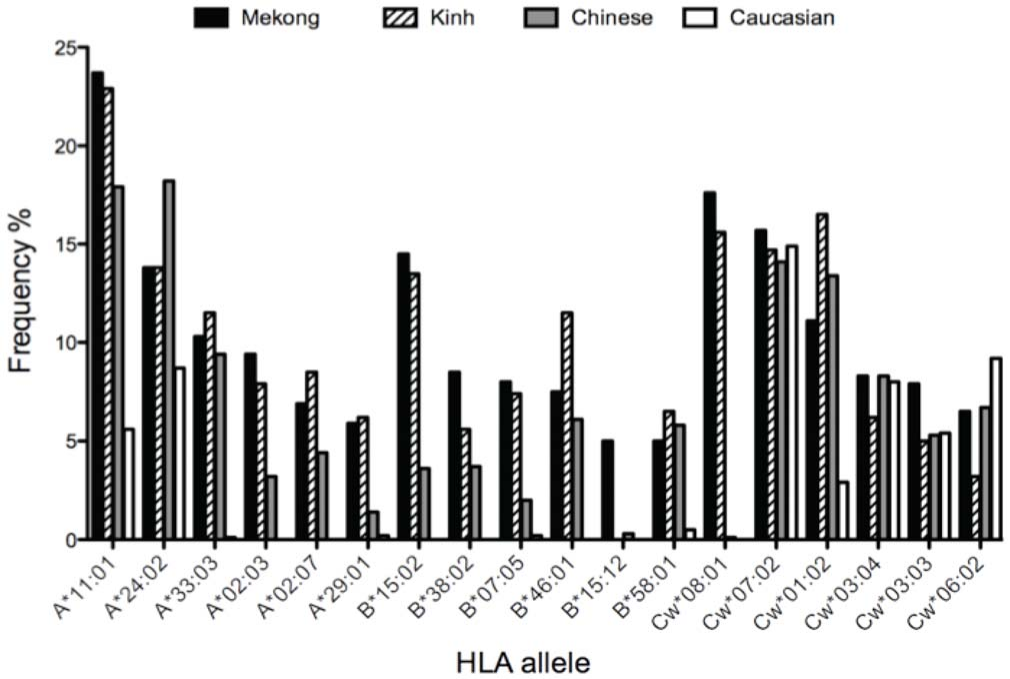
HLA allele frequencies in the Vietnamese Mekong Delta population (n = 116) compared to Kinh Vietnamese (dashed bars), Chinese (grey bars) and European (white bars) populations. The 6 most representative HLA alleles of the Mekong Delta population are represented.

HLA class I typing was performed for 116 patients. The number of different HLA-A, HLA-B and HLA-Cw alleles detected in this study was 21, 42 and 21 respectively. The five most frequent HLA class I alleles in the Mekong population are represented in **Figure 4** and are compared to three other populations: Vietnamese, Chinese and Caucasian people.

The most frequent HLA-A allele was A*11:01, followed by A*24:02, A*33:03 and A*02:03, with a frequency of 23.7%, 13.8%, 10.3%, and 9.4%, respectively. A*02:07 and A*29:01 were also common with a frequency of 6.9% and 5.9%, respectively. The most frequent HLA-B allele was B*15:02, followed by B*38:02, B*07:05 and B*46:01 present in 14.5%, 8.5%, 8% and 7.5% of the population, respectively. B*15:12 and B*58:01 were found at a frequency of 5%. Among HLA Cw alleles, Cw*08:01, Cw*07:02, Cw*01:02, Cw*03:04 and Cw*03:03 were predominant with a frequency of 17.6%, 15.7%, 11.1%, 8.3% and 7.9%, respectively. Moreover, we calculated the linkage disequilibrium in this population and found that most of the pairwise associations were statistically significant with a p value<0.001. The most frequent two-locus haplotypes were B*15:02-Cw*08:01 (23.3%^−^), B*38:02-Cw*07:02 (14.7%) and B*46:01-Cw*01:02 (12.1%). The two most common three-locus haplotypes were A*29:01-B*07:05-Cw*15:04 and A*29:01-B*07:05-Cw*15:05 with a frequency of 9.5%.

### Epitope Processing and HLA-binding predictions

**Figure 5:**
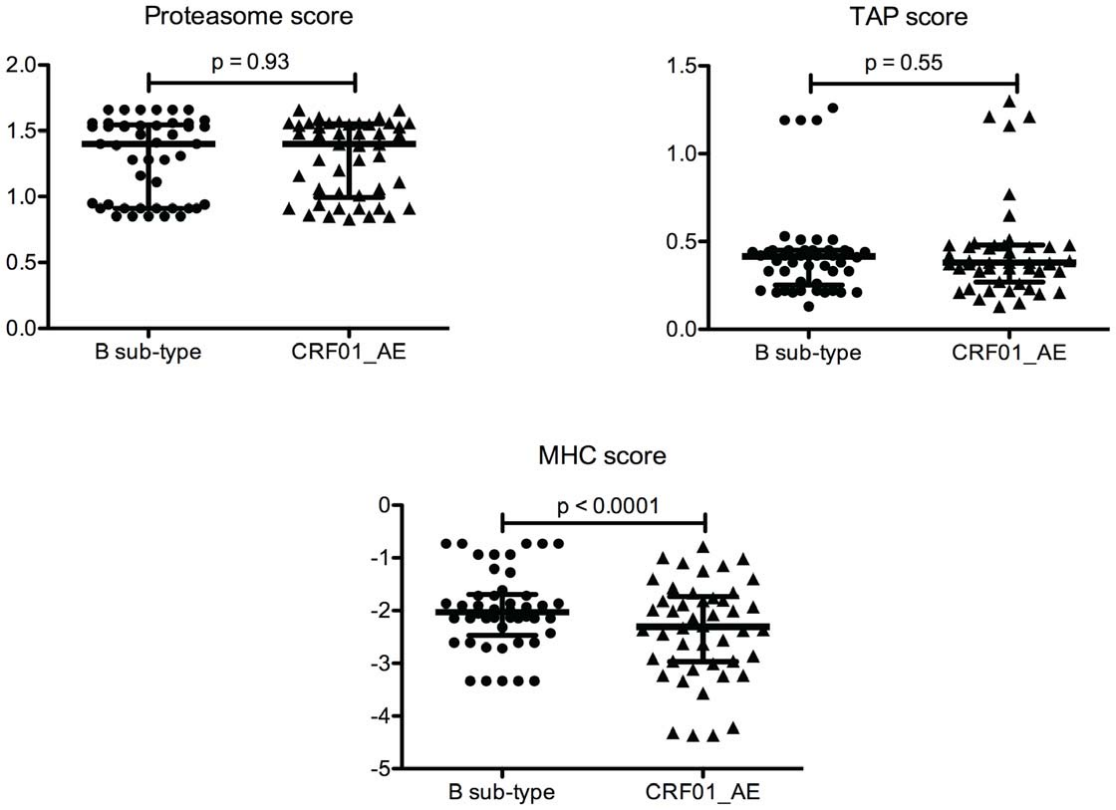
Comparisons of proteasome, TAP and MHC scores between wild-type (B sub-type) and divergent epitopes (CRF01_AE sequences). All scores are logarithmic values, high values corresponding to highly predicted efficiency.

We then investigated amino acid divergences observed within CTL epitopes in the 105 Vietnamese RT-Prot and Gag sequences according to each individual's HLA restriction elements. We found 50 epitopes of interest presenting one to three divergences. The processing into the cell as well as the binding to HLA molecules of each divergent CTL epitope described in the Vietnamese sequences was predicted through the proteasome, TAP and MHC scores (**Figure 5**). The scores for the divergent epitopes were matched and compared with those obtained with the CTL epitopes described for subtype B. The analysis was performed extensively for each HLA restriction element whenever the HLA allele was available on the immune epitope database.

Forty-seven different HLA-restricted epitopes could be analyzed (**Table 3**). Comparisons of the three scores in each group of epitopes are presented in **Figure 4**. Proteasome and TAP scores were comparable in divergent and wild-type epitopes, with a mean (± SD) of 1.29 (±0.3) in each group for the proteasome score and a mean (± SD) of 0.43 (±0.26) for wild-type epitopes *vs* 0.44 (±0.27) for divergent epitopes. The differences were not significant. MHC scores were significantly lower in divergent epitopes with a mean of 2.4 (±0.9) *vs* 2 (±0.7) in wild-type epitopes (p<0.0001). To refine the results, we analyzed the data according to the IC_50_ of each epitope (provided on the IEDB database) based on the rough guideline that peptides with IC_50_ values <50 nM are considered as having high affinity, between 50 and 500 nM as having intermediate affinity and above 500 nM as having low affinity. A strong change in ranking affinity was obtained for 15 epitopes presenting amino acid changes compared to wild-type (highlighted in grey in **Table 3**). The binding affinity evaluated by the MHC score was decreased in 11 out of the 15 epitopes with a mean of −3.18 (±0.85) for the divergent epitopes *vs* −2.04 (±0.57) for the wild-type epitopes (p<0.0001). In 4 out of the 15 epitopes, the MHC score was slightly increased with a mean of −1.6 (±0.15) for the divergent epitopes *vs* −1.57 (±0.31) for the wild-type epitopes (p not significant).

**Table 3:** Proteasome, TAP and MHC score of CTL epitopes.

HLA	Protein location	Wild Type Epitope	Prot. Score	TAP Score	MHC Score	MHC IC_50_[nM]	Mutated Epitope	Prot. Score	TAP Score	MHC Score	MHC IC_50_[nM]
A*24:02	p17	KYKLKHIVW	1,66	0,45	-2,15	142,8	KY**HM**KHIVW	1,6	0,46	−2,15	142
							KY**R**LKHIVW	1,66	0,47	−2,33	212,9
							KY**RM**KHIVW	1,6	0,47	−2,38	237,4
							KY**RM**KH**L**VW	1,56	0,47	−2,08	121
							**Q**YKLKHIVW	1,66	0,38	−2,63	422,3
							**R**Y**RM**KH**L**VW	1,56	0,48	−1,99	98,4
B*35:01	p17	HSSQVSQNY	1,31	1,26	−1,98	95,2	**S**SS**K**VSQNY^b^	1,31	1,3	−3,23	1690,9
B*40:01	p17	IEIKDTKEAL	1,56	0,44	−2,61	402,8	I**D**IKDTKEAL^b^	1,56	0,38	−4,22	16458
							I**DV**KDTKEAL^b^	1,56	0,38	−4,32	20736
							IE**V**KDTKEAL^b^	1,56	0,44	−2,91	819
							I**QV**KDTKEAL^b^	1,56	0,49	−4,37	23631,4
A*02:01	p17	SLYNTVATL	1,54	0,51	−1,72	53	SL**F**N**L**VATL^a^	1,55	0,48	−1,4	25,1
							SL**F**NT**I**ATL^a^	1,55	0,48	−1,57	37
							SL**F**NTVATL^a^	1,54	0,48	−1,66	45,9
A*26:01	p24	EVIPMFSAL	1,41	0,39	−1,21	16,3	EVIPMF**T**AL^b^	1,48	0,39	−1,93	85,5
B*07:02	p24	TPQDLNTML	1,58	0,27	−2,72	526,8	TPQDLNMML	1,57	0,27	−2,96	920,8
B*53:01	p24	QASQEVKNW	1,47	0,36	−2,32	209,7	QA**T**QEVKNW	1,47	0,35	−2,29	193,1
B*57:01	p24	QASQEVKNW	1,47	0,36	−2,43	270,5	QA**T**QEVKNW	1,47	0,35	−2,56	360,5
B*57:01	p24	TSTLQEQIGW	1,4	0,38	−1,94	87,2	TS**N**LQEQIGW^b^	1,4	0,35	−3,24	1719,2
B*58:01	p24	TSTLQEQIGW	1,4	0,38	−1,62	41,4	TS**N**LQEQIGW	1,4	0,35	−2,01	102,3
A*02:01	RT	ALVEICTEM	0,94	0,22	−2,14	138,1	AL**T**EIC**K**EM^b^	1,06	0,17	−2,96	907,6
							AL**I**EICTEM	0,94	0,22	−1,81	64
A*02:01	RT	VIYQYMDDL	1,16	0,53	−2,7	500,1	**I**IYQYMDDL	1,16	0,51	−2,64	439
A*02:01	RT	ILKEPVHGV	0,95	0,13	−2,12	132	ILK**T**PVHGV	1,03	0,13	−2,45	281
A*03:01	RT	AIFQSSMTK	0,91	0,33	−1,28	18,9	AIFQ**C**SMTK	0,91	0,33	−1,4	25,2
A*11:01	RT	AIFQSSMTK	0,91	0,33	−0,94	8,6	AIFQ**C**SM**I**K	0,83	0,33	−1,15	14,1
							AIFQ**C**SMTK	0,91	0,33	−1,1	12,5
							AIFQSSMT**R** b	1,06	0,77	−1,89	77,1
A*11:01	RT	IYQEPFKNLK	0,85	0,21	−3,34	2179,4	IYQEPF**R**NLK	0,86	0,21	−3,23	1699,2
							IYQEPFKNL**R**	1,01	0,65	−4,37	23411
							I**N**QEPFKNLK	0,85	0,15	−3,57	3751,1
							I**F**QEPFKNLK	0,85	0,2	−3,12	1303,6
							IYQE**T**FKNLK	0,85	0,21	−3,34	2209,6
A*11:01	RT	QIIEQLIKK	0,91	0,22	−1,91	81,2	QIIE**E**LIKK	0,91	0,22	−2,01	102,6
							QI**V**E**E**LIKK	0,91	0,23	−2,37	234,4
							Q**V**IE**E**LIKK	0,91	0,23	−2,37	234,4
A*26:01	RT	ETKLGKAGY	1,28	1,19	−1,87	73,6	E**NKQ**GKAGY^b^	1,2	1,16	−3,01	1023,6
							ET**R**LGKAGY	1,28	1,21	−1,82	66,2
							ET**R**LGKAGY	1,28	1,21	−1,82	66,2
B*08:01	RT	GPKVKQWPL	1,11	0,26	−2,06	115	GPKV**R**QWPL^a^	1,11	0,26	−1,76	57,1
B*57:01	RT	IVLPEKDSW	1,39	0,41	−2	100,7	I**E**LPEKDSW^b^	1,39	0,39	−2,86	718,8
B*58:01	RT	IAMESIVIW	1,53	0,42	−0,73	5,4	IA**T**ESI**I**IW	1,48	0,37	−1,02	10,5
							IA**T**ESIVIW	1,53	0,37	−1	10
							I**VT**ESIVIW	1,53	0,38	−1,67	46,4
							IA**I**ESI**I**IW	1,48	0,42	−0,79	6,2
							IA**T**E**G**I**I**IW	1,48	0,37	−1,25	17,6

Wild type epitope scores are matched with the corresponding divergent epitope score (Prot. score = proteasome score).

a Epitopes for which amino acid divergence induces improved MHC binding.

b Epitopes for which amino acid divergence decreases MHC binding.

## Discussion

HIV-1 diversity is modulated at the population level by host immune pressure, which induces a high rate of divergence in immunodominant epitopes recognized by the prevalent HLA class I alleles in these populations. CTL escape mutations occur at critical sites within HLA-restricted CTL epitopes. Indeed, an amino acid substitution may abrogate epitope-HLA binding, reduce T-cell receptor recognition, impair antigen processing or generate antagonistic CTL responses [27]. These mutations result in CTL immune escape but could lead to a severe fitness cost to the virus [28]. Thus, determining the exact kinetics and dynamics of the duel between host and virus is always a challenge. When HIV is transmitted from person to person, mutational escape and reversion rapidly shape HIV evolution. The present global HIV-1 diversity is the result of cumulative infections followed by intra-host viral evolution. The gold standard to analyze it is to provide a comprehensive immunologic and virologic analysis in the context of an acute infection [29]. Due to the difficulty of identifying very early cases of HIV transmission, this type of study is rare.

The present study focused on MHC class I antigen presentation of optimal CTL HIV-1 epitopes across a chronically infected HLA-diverse host population. The patients were HIV-1 infected, all but 3 were ART-naïve, and they all lived in five provincial cities in the Mekong Delta, Vietnam. As expected, our phylogenetic analyses confirmed that HIV-1 CRF01_AE is the unique strain circulating in Southern Vietnam. In addition, DRMs could be detected even in a naive population. The analysis of DRMs on RT-Prot sequences showed 7 ARV-resistant mutants (mostly to NNRTI) among 122 ART-naive patients (6.7%). These results are consistent with a study performed in Northern Vietnam in 2008 showing a prevalence of 4.4% detected for RTI mutations in contrast with a prevalence of 1.7% for PI. This percentage may be explained by the still limited use of PI in Vietnam [19]. We had no information regarding NNRTI use by the corresponding patients for prevention of mother-to-child transmission, which raises the hypothesis that these mutations are SDRMs transmitted from treated to untreated individuals. Since the prevalence is above 5% corresponding to the threshold of surveillance for WHO, our results should be an argument for initiating longitudinal surveys of resistance in the southern part of the country.

The predominance of the CRF01_AE strain is not surprising. Indeed, since the first case of HIV-1 infection detected in Ho Chi Minh City in 1990, the CRF01_AE strain is still largely predominant in Vietnam. Our study is the first to provide data on HIV-1 subtypes in the Mekong area where we identified only the CRF01_AE circulating form. However, there have been a few other studies describing HIV-1 genotypes in other regions of Vietnam such as Ho Chi Minh city, Hanoi and Hai Phong among similar cohorts. In all of them, CRF01_AE predominates although a few other subtypes have been identified such as B' [20] and some recombinants as CRF01_AE/B' [30], CRF02_AG/D [30] and CRF01_AE/C [31].

This wide predominance of CRF01_AE despite individual genetic variability and continuous human influx and efflux remains to be understood. One possible explanation could be a more efficient transmission of the virus. Interestingly, a study in a longitudinal cohort of injection-drug users in Thailand conducted from 1995 through 1998 found an increased probability of transmission of CRF01_AE as compared with subtype B, though it was unclear whether epidemiologic, virologic or host factors were affecting viral spread [32]. In this study, our goal was to evaluate the importance of host factors in the predominance of this viral strain.

The genetic factors of the Vietnamese population living around the Delta Mekong were analyzed according to the distribution of HLA-A, B, and Cw alleles and haplotypes by high-resolution DNA typing. Although there have been some studies on HLA typing in the Vietnamese population, there is only one comprehensive four-digit typing report of HLA class I alleles harbored by the Kinh population in Vietnam, the most prevalent ethnic group in the country [22]. Our results are consistent with the latter study. We clearly show that HLA A*11:01 and B*46:01, which are depicted as Asian alleles, are widely present in our cohort with a high predominance of HLA A*11:01 among HLA-A alleles, whereas the prevalence of both alleles is about 5.6% and 0% in European people, respectively [23]. Other alleles such as A*29:01, B*07:05 and B*15:12 appear to be more specific to the Vietnamese population with a frequency above 5% compared to a frequency below 0.5% in Chinese and European populations [23]. The two major three-digit haplotypes are A*29:01-B*07:05-Cw*15:04 (9,5%) and A*29:01-B*07:05-Cw*15:05 (9,5%), which are considered by Hoa and colleagues as a Kinh signature [22].

Interestingly, even if our population is very similar to the Kinh one, it differs with regard to one specific allele, HLA B*15:12. This allele is absent in the Kinh population and other Vietnamese ethnic populations like the Uyghur [33], Jinua and Wa [34]. It has been described as having a very low frequency (lower than 2.5%) in other Asian populations such as the Chinese, Yi and Hani populations [35]. As this is the first paper reporting such a prevalence for this allele in a defined area, it might be considered as a genetic signature of this population living in the Mekong Delta of Vietnam.

This remarkable diversity and specificity of the HLA class I molecules clearly shape immune responses individually, as the HLA-class I molecules play an important role in CD8+ T-cell recognition. However, the relative impact of these forces on the evolution of HIV at the population level is difficult to evaluate. It has been suggested that virus-clade-specific differences could result, at least in part, from the impact of HLA differences between populations living in distinct regions of the world. Indeed, escape mutations within CD8+ T-cell epitopes can be transmitted relatively frequently and persist in HIV-mismatched recipients, thereby accumulating over time to ultimately represent the most prevalent form of the virus [14], [36], [37], [38].

In our study, we aimed to analyze the impact of divergences within CTL epitopes located in Gag and RT-Prot among Vietnamese HIV-1 strains on HLA class I binding recognition. Results were compared to HLA class I binding recognition of wild-type CTL epitopes from the reference HxB2. Our analysis shows that there is a high degree of polymorphism of CRF01_AE strains compared to the HxB2 reference strain or subtype B strain. Divergences within CRF01_AE CTL epitopes did not modify antigen processing as evaluated by TAP and proteasome scores. However, MHC binding was drastically reduced in two thirds of the divergent epitopes identified, and when the amino acid divergence increased it, the rise was modest. So far, viral escape via mutations within CTL epitopes has been well documented. In most cases, the mutation occurred within the epitope, leading either to reduced binding to the MHC class I molecule and/or an alteration of T-cell receptor recognition. A few mutations affecting antigen processing have also been reported in portable flanking sequences [39].

In our study, all predictions of HLA class I presentation were conducted using T-cell predictions from the Immune Epitope DataBase, but the impact of the divergences we identified on the CTL response could not be assessed, as patients' peripheral blood lymphocytes were not available for such *in vitro* experiments [39]. Therefore, even if these predictions were made using experimental data, they should be interpreted with extreme caution. Proteasome score is the logarithm of the total amount of cleavage site usage releasing the peptide C-terminus. It does not take into account other factors such as the amount of source protein degraded, the type of cell involved in the protein degradation [40] or the amount of immunoproteasome. Moreover, as this software was set up with a limited number of HLA alleles A and B, we could not analyze several alleles that were common in our Vietnamese population, such as: A*33:03, A*02:07, A*29:01, B*38:02, B*07:05 and B*15:12. No data were available for HLA-Cw alleles.

Furthermore, in the absence of *in vitro* analysis, notably ELISpot, compensatory and new CTL responses could not be detected [41] nor could cross-reactive immune responses, as has been shown with subtype A, CRF01_AE and CRF02_AG [42]. Finally, we cannot rule out that the divergences observed in epitopes are chance occurrences, are compensatory, or that they could be due to co-variance and not only result from immune pressure.

However, despite these limitations and in the context of a chronic infection, we demonstrate that in a homogeneous HLA diverse population from the Mekong Delta, Vietnam, MHC binding of known CTL epitopes seems to be strongly reduced due to intraepitopic mutations, thereby facilitating immune evasion of Vietnamese HIV-1 strains. This weak immune pressure combined with a low genetic diversity could explain why CRF01_AE has emerged and spread so rapidly in Southern Asia since 1990.

This finding emphasizes the importance of the immune system in shaping HIV-1 evolution *in vivo* at a population level, as already demonstrated in previous papers. Moore *et al.* elegantly showed that particular host HLA class I alleles are clearly associated with polymorphisms in HIV-1 at sites of least functional or structural constraint, whereas absence of polymorphism is also HLA allele–specific [43]. Other studies suggest that escape mutations within CD8+ T-cell epitopes are frequently transmitted and persist in HIV-mismatched recipients, thereby accumulating over time to ultimately represent the most prevalent form of the virus [37]. More recently, Kawashima *et al.* demonstrated that the frequency of the mutations present in epitopes associated with viral control is strongly correlated with the prevalence of the restricting HLA allele in 9 distinct extensive cohorts [44]. All these data confirm that HIV can adapt to HLA control in order to evade CD8+ T-cell responses and highlight the challenge to find a vaccine to keep pace with the evolving virus.
